# Impact of Examined Lymph Node Count and Lymph Node Density on Overall Survival of Penile Cancer

**DOI:** 10.3389/fonc.2021.706531

**Published:** 2021-07-07

**Authors:** Pan Gao, Tianle Zhu, Jingjing Gao, Hu Li, Xi Liu, Xiansheng Zhang

**Affiliations:** Department of Urology, The First Affiliated Hospital of Anhui Medical University, Hefei, China

**Keywords:** lymph node, examined lymph node count, lymph node density, overall survival, penile cancer

## Abstract

**Background:**

Few studies have explored the optimal examined lymph node count and lymph node density cutoff values that could be used to predict the survival of patients with penile cancer. We further clarify the prognostic value of lymph node density and examined lymph node count in penile cancer.

**Methods:**

The Surveillance, Epidemiology, and End Results (SEER) database was explored to recruit penile cancer patients from 2010 to 2015. A retrospective analysis of penile cancer patients’ data from the First Affiliated Hospital of Anhui Medical University was performed for verification (2006–2016). The cutoff values of examined lymph node count and lymph node density were performed according to the ROC curve. Kaplan-Meier survival analysis was used to compare survival differences among different groups. Univariate and multivariate Cox proportional hazard regression analyses were used to determine the significant variables. On the basis of Cox proportional hazards regression model, a nomogram was established and validated by calibration plot diagrams and concordance index (C-index).

**Results:**

A total of 528 patients in the Surveillance, Epidemiology, and End Results cohort and 156 patients in the Chinese cohort were included in this study. Using the ROC curve, we found that the recommended cutoff values of ELN and LND were 13 and 9.3%, respectively (P <0.001). Kaplan–Meier curves suggested the significant differences of overall survival among different examined lymph nodes and lymph node density. Multivariate analysis indicated ELN and LND were independent prognostic factor for OS of penile cancer patients. Nomogram showed the contribution of ELN and LND to predicting OS was large. The C-index at 3-, and 5-year were 0.744 for overall survival (95% CI 0.711–0.777).

**Conclusions:**

The more lymph nodes examined, the lower the density of lymph nodes, and the higher the long-term survival rate of penile cancer. We recommended 13 examined lymph nodes and lymph node density >9.3% as the cutoff value for evaluating the prognosis of penile cancer patients.

## Introduction

Penile cancer (PeCa) is a rare disease, but its incidence has been rising slowly in recent years. According to the 2020 Cancer Research UK (CRUK) report, the incidence rate has increased by 15% over the past decades (1).

As we all know, PeCa is an aggressive urological malignancy, which follows the pattern of gradual invasion from the primary tumor site to inguinal lymph nodes (LNs) before its systemic spread (2, 3). Previous studies have shown that nodal involvement is the most important prognostic factor in PeCa (4). Patients with pN2 and pN3 stages have a 5-year cancer specific survival ranging from 17 to 60% and 0–17%, respectively (5). Although according to the current research on the TNM staging of PeCa, the number of positive LNs can predict the overall survival (OS), like other tumors, the resection quantity of LN metastasis is affected by various factors in survival analysis, such as LN resection method, pathologist’s evaluation and individual physiological changes, these mask the true degree of LN involvement to a certain extent (6–9). Therefore, a more optimized variable is needed to evaluate the OS.

From the previous studies we have known that examined lymph node (ELN) count and lymph node density (LND) are the percentage of positive LNs, which have been used as a prognostic factor for other tumors, such as esophageal cancer, non-small-cell lung cancer and bladder cancer (2, 10–14). Unfortunately, these were rarely studied in PeCa. A study conducted by Li et al. determined the prognostic value of ELN in patients with PeCa, but the number of patients was relatively small (6). Additionally, Pettaway et al. first reported the significance of LND for PeCa in 2009 and also, the European Urological Association (EAU) recommended LND for the first time to predict the prognosis of PeCa patients in 2014 (15, 16). However, they didn’t calculate the exact optimal cutoff value.

Nomogram, a statistical forecasting tool, has the advantages of low cost and strong reliability, which is used to quantify individual risks according to forecasting factors (4, 17). However, nomogram for predicting the survival of penile cancer patients is rarely constructed. Zheng et al. developed a nomogram that incorporated age, N classification, and log odds of positive LNs which could be conveniently used to predict the long-term OS of patients with penile squamous cell carcinoma (18). However, the variable of ELN and LND was not included in their study.

Therefore, in the current study, we analyzed the effect of ELN and LND on OS in patients with PeCa and evaluated the extent of this effect. Moreover, we included the variable ELN and LND to create an accurate and personalized prognostic nomogram for predicting OS in patients with PeCa, in order to further clarify the prognostic value of ELN and LND in PeCa.

## Materials And Methods

### Study Design and Data Source

This is a retrospective study, using the clinical data of two groups of people diagnosed with PeCa: one from the Surveillance, Epidemiology, and End Results (SEER) database as the training cohort (1975–2016) and the other from Blinded for peer review of China as the validation cohort (2006–2016). All patients in both cohorts underwent radical lymphoadenectomy in addition to surgery of primary tumor site. In patients with nonpalpable nodes, a superficial dissection above the fascia lata was performed. In cases with palpable adenopathy or suspicious nodes encountered during superficial dissection, a deep dissection was performed. Pelvic lymphadenectomy was performed in patients with positive deep inguinal lymph nodes or with enlarged pelvic lymph nodes on cross sectional imaging. The demographic information of age at diagnosis, marital status at diagnosis, ELN, LND, surgery of primary site and tumor characteristics of differentiation grade, histological type, T-stage, N-stage, M-stage and tumor size were collected. Incompletely documented variables such as primary surgical site, grade, TNM stage, marital status, tumor size, ELN, and positive lymph nodes were excluded from this study. In the calculation of “examined lymph node count” and “lymph node density”, inguinal and pelvic lymph nodes were included.

OS is defined as the time from diagnosis to original death, whatever the reasons. TNM staging and histopathological grading of PeCa were determined according to the American Joint Committee on Cancer (AJCC) 6th edition staging system and SEER cancer grading system, respectively.

The SEER database is a publicly available, federally funded cancer reporting system and also the largest publicly available cancer data set. Institutional review committees and ethics committees allow the use of public database data without patient identity information (19). Additionally, this study was approved by our University Research Subject Review Board.

### Statistical Methods

All statistical analyses were performed using SPSS version 20.0 (SPSS Inc, Chicago, IL, USA). Chi-square, Pearson’s chi-square, and Fisher’s exact tests were used to determine the significance of differences between continuous variables and categorical variables. Kaplan–Meier analysis was used to estimate survival and compare different variables, namely, average survival time, median survival time and 95% confidence interval (95% CI). Based on Cox proportional hazard regression analysis, multivariate and univariate survival analyses were conducted. As for the evaluation of the model performance and the verification of the accuracy of the new scoring system, we use the Harrell concordance index (C-index) and calibration curve, respectively. Moreover, the receiver operating characteristic (ROC) curve was used to evaluate the effectiveness of the nomogram. *P <*0.05 values were considered statistically significant for all.

## Results

### Cutoff Values of ELN and LND

**Figure 1:**
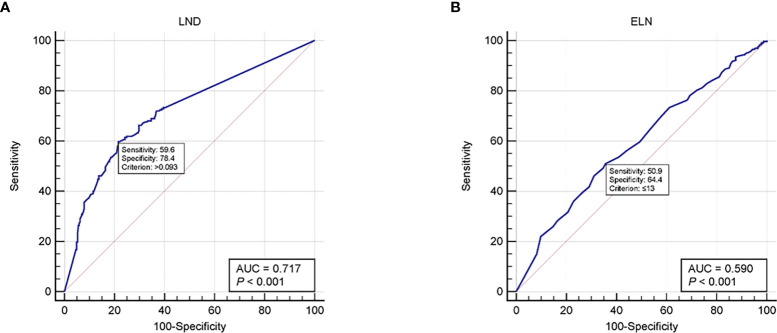
ROC curves for **(A)** LND and **(B)** ELN. ROC, Receiver operating characteristic; LND, Lymph node density; ELN, Examined lymph node.

At present, in clinical diagnostic trials, an ROC curve is used to select the critical value reasonably. The curve area under the optimal critical point is the largest, its sensitivity and specificity are relatively high, and the number of misdiagnosis and missed diagnosis is also the smallest. Using the ROC curve, we found that the recommended cutoff values of ELN and LND were 13 [sensitivity, 50.9; specificity, 64.4; AUC (area under the ROC curve), 0.59; *P <*0.001] and 9.3% [sensitivity, 59.6; specificity, 78.4; AUC, 0.717; *P <*0.001], respectively (**Figure 1**).

### Patient Characteristics

After screening, 528 patients in the SEER cohort and 156 patients in the China cohort were included in this study. As shown in **Table 1**, all variables had no statistical difference between the training group and the validation group (*P >*0.05 for all).

**Table 1:** Demographics and clinicopathological characteristics of patients in training cohort and validation cohort.

Demographics and clinicopathologic characteristics	Training set (n = 528)		Validation set (n = 156)		*P*-value
	No. of patients	%	No. of patients	%	
Age at diagnosis (year)					0.825
<50	96	18.2	25	16.0	
50–69	271	51.3	82	52.6	
≥70	161	30.5	49	31.4	
Marital status					0.877
Married	306	60.0	85	54.5	
Divorced	64	12.1	21	13.5	
Widowed	39	7.4	14	9.0	
Single	101	19.1	32	20.5	
Unknown	18	1.4	4	2.5	
Grade					0.763
G1	73	13.8	23	14.7	
G2	283	53.6	82	52.6	
G3	142	26.9	46	29.5	
G4	7	1.3	1	0.6	
Unknown	23	4.4	4	2.6	
T-stage					0.656
T1	138	26.1	41	26.3	
T2	219	41.5	59	37.8	
T3 + T4	171	32.4	56	35.9	
N-stage					0.793
N0	238	45.1	75	48.1	
N1	115	21.8	30	19.2	
N2	115	21.8	36	23.1	
N3	60	11.3	15	9.6	
M-stage					0.477
M0	510	96.6	149	95.5	
M1	18	3.4	7	4.5	
Histological type					0.804
SCC	491	93.0	144		
PC	15	2.8	5		
LC	18	3.4	7		
BCC	1	0.2	0	92.3	
TCC	3	0.6	0	7.7	
ELN					0.854
≤13	221	41.9	67	42.9	
>13	307	58.1	89	57.1	
LND					0.707
≤9.3%	328	62.1	100	64.1	
>9.3%	200	37.9	56	35.9	
Tumor size					0.467
≤3.5 cm	285	54.0	79	50.6	
>3.5 cm	243	46.0	77	49.4	
Surgery of primary site					0.927
LTE	60	11.4	18	11.5	
SS	432	81.8	126	80.8	
RS	36	6.8	12	7.7	

SCC, Squamous cell carcinoma; PC, Papillary carcinoma; LC, Lymphoepithelial carcinoma; BCC, Basal cell carcinoma; TCC, Transitional cell carcinoma; LTE, Local tumor excision; SS, Simple/partial surgical removal of primary site; RS, Radical surgery; ELN, Examined lymph node; LND, Lymph node density.

### Relationship Between LND and Demographics/Clinicopathologic Characteristics

With the cutoff value obtained by ROC curve, we divided all the patients of the training group into two groups: LND ≤rain and LND >9.3%, the numbers were 328 (62.1%) and 200 (37.9%), respectively. The connection is displayed in **Table 2**. LND wasn’t significantly correlated with marital status (*P* = 0.6); however, the association between LND and age at diagnosis (*P* = 0.003), grade (*P <*0.001), T-stage (*P* = 0.001), N-stage (*P <*0.001), M-stage (*P* = 0.002), histological type (*P* = 0.007), ELN (*P <*0.001), tumor size (*P* = 0.012) and surgery of primary site (*P* = 0.012) were significant.

**Table 2:** Relationship between LND and demographics/clinicopathologic characteristics.

Demographics/clinicopathologic characteristics	LND (n = 528)				*P*-value
	≤9.3% (n = 328)		>9.3% (n = 200)		
	No. of patients	%	No. of patients	%	
Age at diagnosis (year)			35	17.5	0.003
<50	61	18.6	87	43.5	
50–69	184	56.1	78	39.0	
≥70	83	25.3			
Marital status					0.6
Married	189	57.6	117	58.5	
divorced	35	10.7	29	14.5	
widowed	25	7.6	14	7.0	
single	68	20.7	33	16.5	
unknown	11	3.4	7	3.5	
Grade					0.000
G1	61	18.6	12	6.0	
G2	182	55.5	101	50.5	
G3	67	20.4	75	37.5	
G4	2	0.6	5	2.5	
Unknown	16	4.9	7	3.5	
T-stage					0.001
T1	93	28.4	45	22.5	
T2	148	45.1	71	35.5	
T3 + T4	87	26.5	84	42.0	
N-stage					0.000
N0	238	72.6	0	0.0	
N1	52	15.9	63	31.5	
N2	27	8.2	88	44.0	
N3	11	11.3	49	24.5	
M-stage					0.002
M0	323	98.5	187	93.5	
M1	5	1.5	13	6.5	
Histological type					0.007
SCC	300	91.5	191	95.5	
PC	13	4.0	2	1.0	
LC	15	4.5	3	1.5	
BCC	0	0.0	1	0.5	
TCC	0	0.0	3	1.5	
ELN					0.000
≤13	109	33.2	112	56.0	
>13	219	66.8	88	44.0	
Tumor size					0.012
≤3.5 cm	191	58.2	94	47.0	
>3.5 cm	137	41.8	106	53.0	
Surgery of primary site					0.012
LTE	38	11.6	22	11.0	
SS	276	84.1	156	78.0	
RS	14	4.3	22	11.0	

SCC, Squamous cell carcinoma; PC, Papillary carcinoma; LC, Lymphoepithelial carcinoma; BCC, Basal cell carcinoma; TCC, Transitional cell carcinoma; LTE, Local tumor excision; SS, Simple/partial surgical removal of primary site; RS, Radical surgery; ELN, Examined lymph node; LND, Lymph node density.

### Comparison of Oncology Features of Patients With Different LND

**Figure 2:**
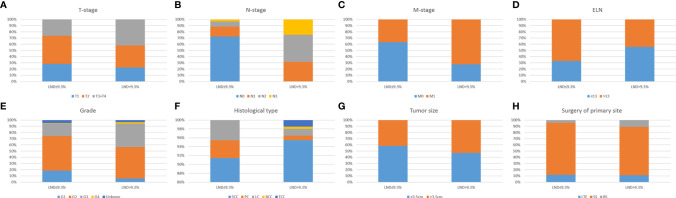
Comparison of oncology features of penile cancer patients with different LND. Significant differences in the distribution of T-stage **(A)**, N-stage **(B)**, M-stage **(C)**, ELN **(D)**, Grade **(E)**, Histological type **(F)**, Tumor size **(G)**, Surgery of primary site **(H)**, with different LND (all *P < *0.05). LND, Lymph node density; ELN, Examined lymph node.

Patients were divided into groups according to LND, and the oncology characteristics of each group were compared (shown in **Figure 2**). There are significant differences in the distribution of T-, N-, and M-stages, histological type, tumor grade and size among different LND patients (*P <*0.05 for all). Generally speaking, LND is closely related to the pathological features of tumors.

### Distribution and Correlation of Clinicopathological Features of Patients

**Figure 3:**
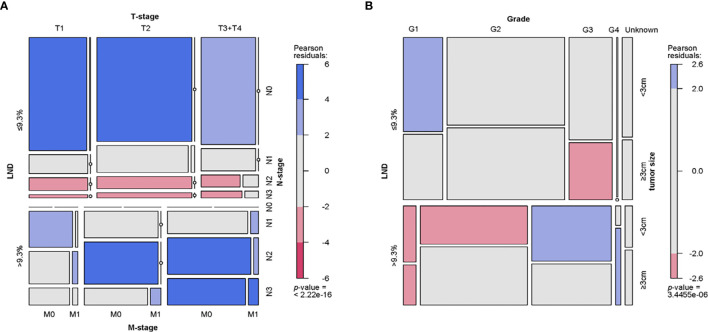
Mosaic plot. **(A)** Distribution and relationship of LND, T-stage, N-stage and M-stage. **(B)** Distribution and relationship of LND, tumor grade, and tumor size. LND, Lymph node density.

The distribution and correlation of clinical and pathological characteristics of patients in the training group were represented by the mosaic plot to which area of the nested matrix is proportional to the unit frequency, and the frequency is the frequency in the multi-dimensional contingency table. The residual value of fitted model are represented by color and shading. Patients with LND >9.3% have the characteristics of higher tumor grade, more prone to distant metastasis, higher clinical tumor stage and larger tumor size. Also, their histopathological types are significantly different from LND (**Figure 3**).

### Univariate and Multivariate Analyses and Identification of Predictors of OS

Univariate risk factors of OS are shown in **Table 3**. We can see that age at diagnosis, marital status, grade, N- and M-stages, surgery of primary site, tumor size, ELN and LND were significant prognostic factors. Besides, as indicated by multivariate analysis, age at diagnosis, N- and M-stages, ELN and LND were independent prognostic factors for OS.

**Table 3:** Univariate and multivariate analysis of the training cohort.

Variables	Univariate analysis		Variables	Multivariate analysis	
	HR (95% CI)	p-value		HR (95% CI)	p-value
**Statistically significant factors**			**Statistically significant factors**		
Age at diagnosis (years)			Age at diagnosis (years)		
<50 *vs.* 50–59	1.009 (0.686–1.486)	0.961	<50 *vs.* 50–59	1.093 (0.733–1.631)	0.661
<50 *vs.* ≥70	1.702 (1.144–2.531)	0.009	<50 *vs.* ≥70	1.637 (1.066–2.514)	0.024
Marital status at diagnosis			N-stage		
Married *vs.* divorced	1.332 (0.899–1.975)	0.152	N0 *vs.* N1	1.904 (1.182–3.069)	0.008
Married *vs.* widowed	1.663 (1.066–2.596)	0.024	N0 *vs.* N2	1.960 (1.143–3.362)	0.014
Married *vs.* single	1.198 (0.847–1.693)	0.305	N0 *vs.* N3	4.045 (2.303–7.103)	<0.001
Married *vs.* unknown	0.419 (0.133–1.317)	0.136	M-stage		
Grade			M0 *vs.* M1	2.154 (1.212–3.826)	0.009
G1 *vs.* G2	1.197 (0.792–1.809)	0.391	ELN		
G1 *vs.* G3	1.740 (1.121–2.700)	0.013	≤13 *vs.* >13	0.718 (0.524–0.983)	0.039
G1 *vs.* G4	1.421 (0.431–4.679)	0.562	LND		
G1 *vs.* Unknown	0.772 (0.350–1.700)	0.521	≤9.3% *vs.* >9.3%	1.903 (1.218–2.974)	0.005
N-stage			**Statistically non-significant factors**		
N0 *vs.* N1	2.874 (2.010–4.109)	<0.001	Marital status at diagnosis		
N0 *vs.* N2	3.081 (2.148–4.418)	<0.001	Married *vs.* divorced	1.197 (0.797–1.798)	0.387
N0 *vs.* N3	5.851 (3.955–8.654)	<0.001	Married *vs.* widowed	1.443 (0.903–2.306)	0.125
M-stage			Married *vs.* single	1.165 (0.810–1.677)	0.410
M0 *vs.* M1	3.558 (2.062–6.138)	<0.001	Married *vs.* unknown	0.457 (0.142–1.472)	0.190
Surgery of primary site			Grade		
LTE *vs.* SS	1.1040 (0.721–1.691)	0.649	G1 *vs.* G2	0.760 (0.489–1.181)	0.222
LTE *vs.* RS	2.207 (1.260–3.867)	0.006	G1 *vs.* G3	0.813 (0.496–1.333)	0.412
ELN			G1 *vs.* G4	0.696 (0.203–2.380)	0.563
≤13 *vs.* >13	0.644 (0.470–0.836)	0.001	G1 *vs.* Unknown	0.493 (0.221–1.103)	0.085
LND			Surgery of primary site		
≤9.3% *vs.* >9.3%	0.261 (0.200–0.342)	<0.001	LTE *vs.* SS	1.028 (0.644–1.640)	0.909
Tumor size			LTE *vs.* RS	1.467 (0.807–2.668)	0.209
≤3.5 cm *vs.* >3.5 cm	1.421 (1.095–1.844)	0.008	Tumor size		
**Statistically non-significant factors**			≤3.5 cm *vs.* >3.5 cm	1.237 (0.936–1.636)	0.135
Histological type					
SCC *vs.* PC	0.386 (0.123–1.205)	0.101			
SCC *vs.* LC	0.812 (0.360–1.829)	0.615			
SCC *vs.* BCC	0 (0.000–7.615E+102)	0.943			
SCC *vs.* TCC	0.867 (0.121–6.195)	0.887			
T-stage					
T1 *vs.* T2	0.847 (0.609–1.179)	0.326			
T1 *vs.* T3 + T4	1.251 (0.898–1.744)	0.184			

SCC, Squamous cell carcinoma; PC, Papillary carcinoma; LC, Lymphoepithelial carcinoma; BCC, Basal cell carcinoma; TCC, Transitional cell carcinoma; LTE, Local tumor excision; SS, Simple/partial surgical removal of primary site; RS, Radical surgery; ELN, Examined lymph node; LND, Lymph node density.

### Kaplan–Meier Survival Analysis for Different LND/ELN

**Figure 4:**
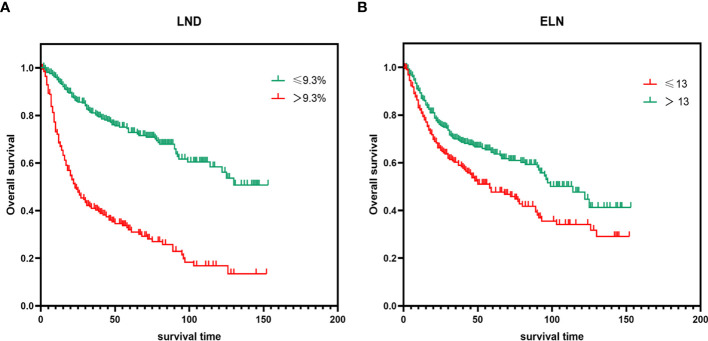
Kaplan–Meier survival analysis for different **(A)** LND and **(B)** ELN (*P < *0.001 for all). LND, Lymph node density; ELN, Examined lymph node.

In order to evaluate the OS of PeCa patients with different LND/ELN, the Kaplan–Meier survival analysis was performed on all patients. As shown in **Figure 4**, the significant differences of OS were seen among different LND/ELN (*P* < 0.001 for all). Patients with LND ≤9.3% had the highest OS (median OS and 95%CI undefined), followed by LND >9.3% (median OS = 23, 95%CI = 16.565–29.435). Similarly, patients with ELN >13 have the highest survival rate (median OS = 114, 95%CI = 88.966–139.034), followed by ELN ≤39 (median OS = 58, 95%CI = 36.546–79.454).

### Construct and Validate Nomogram

**Figure 5:**
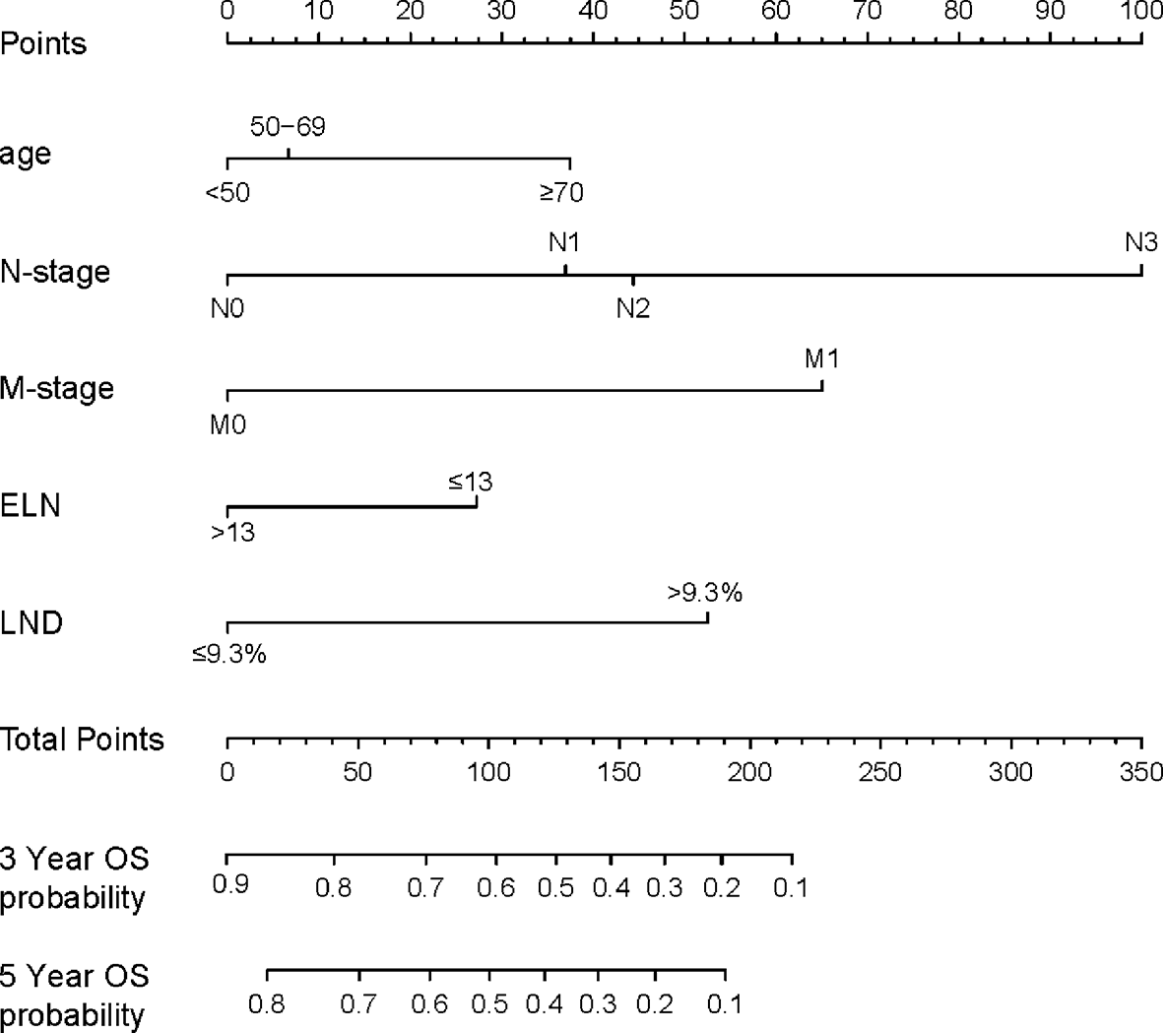
A nomogram for predicting the OS. In order to use the nomogram, the value of each predicted value is determined by drawing a line up to the point reference line, these points are summed, and drawing a line down from the total point line to find the predicted probability of OS. OS, Overall survival; LND, Lymph node density; ELN, Examined lymph node.

On the basis of Cox proportional hazards regression model, age, N- and M-stages, ELN and LND were selected as variables to construct nomogram (**Figure 5**). Each variable has a corresponding score from 0 to 100 according to its contribution to the result variable. Then add the scores to get the total score at the bottom, and finally calculate the predicted value of the individual outcome event through the functional transformation relationship between the total score and the probability of occurrence of the outcome event. From the nomogram, we know the selected factors had varying degrees of influence on OS. The nomogram scoring system is displayed in **Table 4**.

**Table 4:** Nomogram scoring system.

Variables	Points	Variables	Points
Age at diagnosis (years)		M-stage	
<50	0	M0	0
50–69	7	M1	65
≥70	38	ELN	
N-stage		≤13	28
N0	0	>13	0
N1	38	LND	
N2	45	≤9.3%	0
N3	100	>9.3%	53
3-Year OS probability	Points	5-Year OS probability	Points
0.1	218	0.1	191
0.2	190	0.2	164
0.3	169	0.3	142
0.4	148	0.4	121
0.5	128	0.5	100
0.6	102	0.6	78
0.7	78	0.7	50
0.8	41	0.8	28
0.9	0		

ELN, Examined lymph node; LND, Lymph node density; OS, Overall survival.

**Figure 6:**
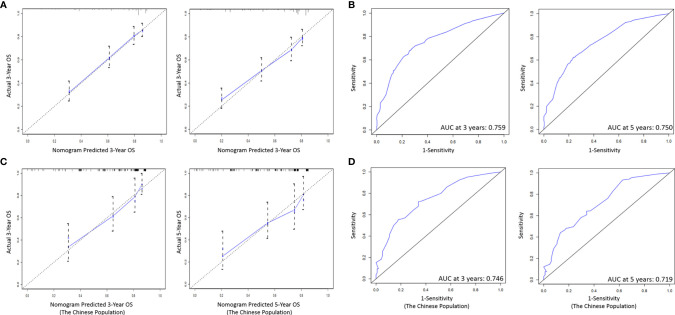
**(A)** Calibration curves of the prognostic nomogram for 3-, and 5-year OS in the training set. **(B)** The ROC curve of the prognostic nomogram in the training set. **(C)** Calibration curves of the prognostic nomogram for 3-, and 5-year OS in the validation set. **(D)** The ROC curve of the prognostic nomogram in the validation set. ROC, Receiver operating characteristic; OS, Overall survival.

As shown in **Figure 6A**, the ability of the model to predict the 3- and 5-year OS of PeCa patients was verified by the calibration curve (C-index value: 0.744 >0.7, suggesting that our nomogram is suitable for patients with PeCa). To further validate the performance of the model, the ROC curve was plotted for the nomogram (**Figure 6B**), and the AUC of the nomogram was large, which shows that the accuracy of nomogram was good.

### Verified by External Population

On the basis of the nomogram, we drew 3- and 5-year calibration curves and ROC curves from our single center population for independent verification, and the results of the curves were in high agreement with the results of our training group (**Figures 6C, D**).

## Discussion

Previous studies have shown that LN status is the most important prognostic factor of PeCa, and its influence on the prognosis of the disease is more significant than that of the tumor grade, general appearance, morphology or microscopic pattern of the primary tumor (20–23). ELN and LND are two basic aspects to determine the status of LNs, which are considered to be predictive factors for the survival of patients with other types of cancer (7, 24). However, up to now, there is no suggestion about ELN count in the National Comprehensive Cancer Network (NCCN) PeCa Guide, although some studies tried to set a benchmark, and the results are not satisfactory (16, 25–27). Recently, Mao et al. used multivariate Cox regression analysis to show that ≥ RLNs removed indicates lower all-cause mortality, PeCa-specific mortality, and lower 5-year mortality, but they had no data to indicate why the cutoff value of the removed LN was 8 (28). Another study conducted by Li et al. reported that the removal of at least 16 lymph nodes in PeCa patients is related to the significant prolongation of disease-specific survival rate, however, they did not have any data on the correlation between the number of LNs removed and OS (6).

Of note, as illustrated in our study, we not only show that ELN is an independent predictor of survival of PeCa, but also that OS with ELN >13 are significantly higher than OS with ELN ≤13. The key point is that we calculate the appropriate threshold for ELN is 13. This shows from another perspective that the more LNs are examined, the less positive LNs are not detected, and this may lead to more thorough removal of remnants to improve long-term survival. Therefore, in PeCa patients with positive and negative LN status, the more the number of LNs examined, the higher the OS, and there is a consistent positive correlation between them.

Additionally, previous studies have shown that the burden of LNs expressed by the number of positive LNs is related to poor prognosis (29, 30). Compared with the number of positive LNs, LND is a more optimized index in the prognosis of PeCa, which can reflect both the degree of LN dissection and the disease burden of LNs (2, 9). The significance of the LND for PeCa was first reported by Pettaway et al. in 2009. In their study, they proved that LND is a better index to predict the disease specific survival of PeCa than the TNM LN staging system (15). Subsequently, in 2014, LND was first recommended by EAU to predict the prognosis of PeCa patients (16). However, in limited studies, the critical value of optimal LND varies widely, ranging from 6.7 to 33% (6, 10, 31). Unlike previous studies, in our study, we not only conformed that LND is a predictor of PeCa, but also, we determined that the recommended cutoff value for LND is 9.3%. More significantly, we found that LND has a good predictive significance for OS in the nomogram and it is verified by external data.

In recent years, nomogram, as a statistical model, shows high reliability in predicting tumor progression (32). Zheng et al. established a simple nomogram for predicting OS for the first time by using the cohort of contemporary penile squamous cell carcinoma patients from the SEER database, in which only three variables were integrated, including age, nitrogen classification and log odds of positive LNs in 2020 (18). Svatek et al. also conducted similar research; they stratified survival outcomes simply according to its median LND of 6.7%, which limits its clinical applicability (15). So far, no studies have included ELN and LND to build nomogram to predict OS of PeCa. Our research indicates that the following five factors are independently related to OS of PeCa patients, including age, N- and M-stages, ELN and LND. All the above factors are included in the construction of the nomogram. As seen in our nomogram, LND contributes more to prognosis than ELN, suggesting that LND has better prognostic value than ELN.

To our knowledge, our study was the first to thoroughly examine the prognostic role of ELN and LND in PeCa and to develop a nomogram to predict its impact on the OS. What is important is that we use real-world data sets with reliable statistics for verification. We sought to emphasize two major points: (I) ELN and LND are independent predictors for survival of PeCa. (II) A greater number of ELNs and lower LND are associated with better long-term survival of PeCa. We recommended 13 ELNs and LND >9.3% as the cutoff value for evaluating the prognosis of PeCa patients. Therefore, surgeons and pathologists should try their best to explore the LNs and the minimum recommended number for assessing the integrity of LN sampling is 13 and LND needs to be at least 9.3%. Based on real patient data, our research emphasize that surgeons should fully sample and dissect LNs in clinical practice, and carefully explore LNs.

Due to the limitation of retrospective and small-scale real data, the prognostic significance of our results may be discounted a little. First, the main limitation is that the universality of our study may be limited by the fact that it is conducted in a single cultural/social context. Our research is carried out in one country, which is probably a relatively homogeneous population. Due to the lack of sample size and stratified sampling, it cannot represent the true situation of all PeCa patients, and the results will inevitably be influenced by local culture. Therefore, this research needs to be carried out in more countries and regions. Second, the results may still be affected by the selection bias inherent in the design of this study, because adjuvant therapy (including adjuvant chemotherapy and/or radiotherapy) and pelvic lymphadenectomy may affect other parameters. Third, we were unable to investigate other important issues, such as the influence of the number of LNs at stations N1 and N2. As the treatment of PeCa progresses, the prognostic significance of our ELN and LND cut-off values may be changed, so this finding needs to be verified in other cohorts. Fourth, SEER databases may include inhomogeneous data about data collection deriving also from different intern protocols adopted by each center enrolled patients coming from.

Despite these limitations, our analysis demonstrates that the greater the number of LNs examined, the smaller the LND value, and the higher the long-term OS of patients with PeCa. We recommend checking at least 13 LNs and LND >9.3% as a cut-off point for assessing the prognostic stratification of patients with PeCa. This further proves that ELN and LND are tools for predicting PeCa. More institutional research is needed to further determine the clinically relevant prognosis data of the disease.

## Data Availability Statement

The raw data supporting the conclusions of this article will be made available by the authors, without undue reservation.

## Author Contributions

PG and XZ designed the study. JG provided the databases. PG, TZ, HL, and XL assembled and analyzed the data. PG wrote the manuscript. All authors contributed to the article and approved the submitted version.

## Funding

This study was funded by the National Natural Science Foundation of China (No. 82071637) and Natural Science Foundation of Anhui Province, China (No. 2008085QH420).

## Conflict of Interest

The authors declare that the research was conducted in the absence of any commercial or financial relationships that could be construed as a potential conflict of interest.
